# Distichiasis-lymphedema syndrome with optic disc pit

**DOI:** 10.4103/0301-4738.73703

**Published:** 2011

**Authors:** K Kaarthigeyan, M Ramprakash, G Kalpana

**Affiliations:** Department of Pediatrics, PSG Institute of Medical Sciences and Research, Coimbatore, India; 1 Department of Ophthalmology, Kanchi Kamakoti CHILDS Trust Hospital, Chennai, India; 2 Department of Clinical Genetics, Kanchi Kamakoti CHILDS Trust Hospital, Chennai, India

Dear Editor,

An eight-year old boy, first born to third degree consanguineous parents, presented with right leg swelling for three months, with gradual onset, which progressed up to knee. There was no history of fever, injury, abdominal pain or contact with tuberculosis. He was treated with anti-filarial drugs elsewhere. At two years of age, he had frequent episodes of redness and constant rubbing of eyes and was then diagnosed to have double-rowed eye lashes involving all four eyelids and the extra rows of lashes were cauterized and removed elsewhere. The boy still continued to be symptomatic. None of the other family members had similar complaints.

**Figure 1:**
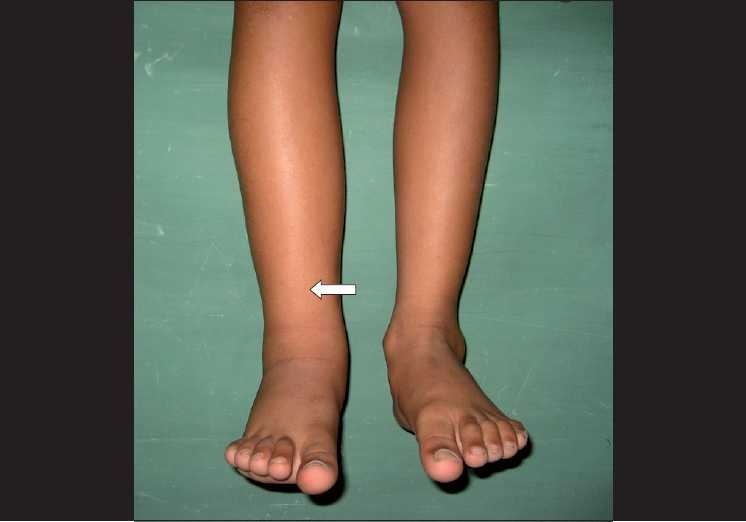
‘Lymphedema’ of the right lower limb, confined to below-knee level and asymmetric

**Figure 2:**
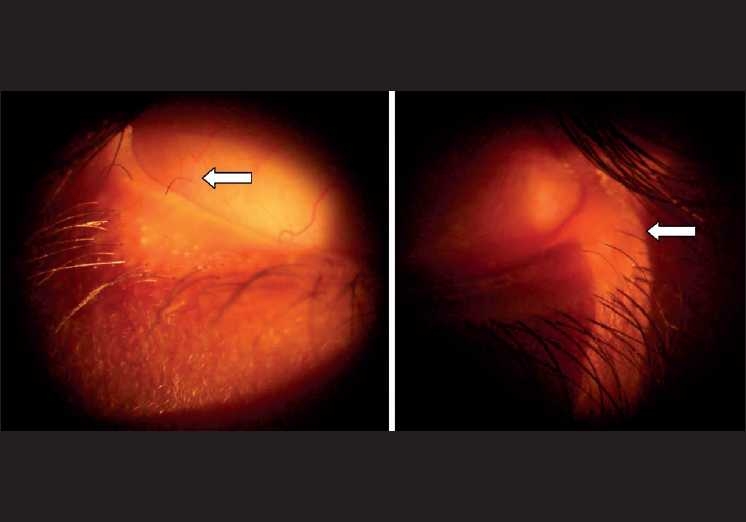
‘Distichiasis’ – aberrant eyelashes arising from the meibomian glands on the inner aspects of the upper and lower eyelids. These can range from a full set of extra eyelashes to a single/ few hair

**Figure 3:**
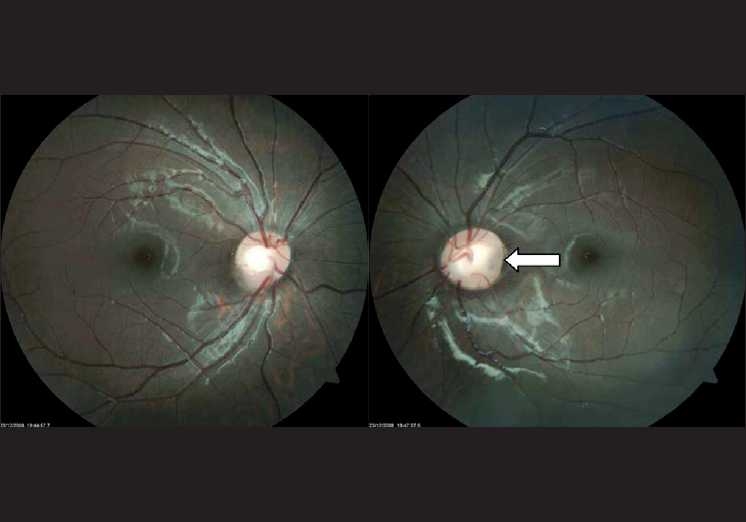
‘Optic disc pit’ in the left eye – found in the temporal part of the optic nerve head and the disc on the affected eye was larger than the fellow disc

On examination, he had right lower limb edema, which was from the knee downward. [Fig. 1] There were no bony deformities or vertebral anomalies. Systemic examination was normal. He had mild congestion of both eyes. His visual acuity was 20/20; N6 in both eyes, and had no refractory error. Slit-lamp examination revealed distichiasis. [Fig. 2] A focal area of loss of eyelashes and depigmentation of skin was noted in the left upper eyelid. Fundus examination revealed an optic disc pit in the left eye and the macula was normal [Fig. 3].

Blood parameters were normal. Night smears for microfilaria were negative. Ultrasonography (USG) abdomen, echocardiogram, magnetic resonance imaging (MRI) spine, and vascular Doppler studies of both limbs were normal. Isotope lymphoscintigraphy confirmed the lymphedema. The parents were also screened and found to be normal. A clinical diagnosis of distichiasis-lymphedema syndrome (DLS) was made. Conservative management for symptomatic distichiasis, with lubrication and epilation was carried out, advice for Amsler test at home periodically and stockings for lymphedema were given. The parents were genetically counseled for prevention of secondary complications such as, cellulitis, foot infections, and varicose veins.

Lymphedema in DLS typically appears in late childhood/ puberty. It is confined to the lower limbs, usually bilateral and often asymmetric, becoming evident between 5 and 20 years of age.[1,2] Distichiasis presents from an early age, probably at birth, where accessory eyelashes occur along the posterior border of the lid margins in the position of the Meibomian gland orifices.[2] It is associated with irritative ocular problems namely corneal irritation, recurrent conjunctivitis, and photophobia.[1] The extra eyelashes can usually be seen on torch light examination, but in some cases slit lamp examination is required.

DLS has an autosomal dominant inheritance pattern with marked variability of expression. Mutations in the Forkhead family gene FOXC2 located on chromosome 16q24.3 have been identified to be associated with this syndrome.[3]

Epidural cysts, cardiac abnormalities, short stature, ptosis, microphthalmia, strabismus, partial ectropion of the lower lid, pterygium coli, chylothorax, cleft palate, bifid uvula, micrognathia, scoliosis/ kyphosis, and cryptorchidism are the other occasional abnormalities reported with DLS.[1]

The optic disc pit is a congenital anomaly characterized by excavation of the optic nerve head and is usually unilateral. It is commonly found in the temporal part of the optic nerve head and the disc on the affected eye is larger than the fellow disc.[4] Patients with optic disc pit may develop complications like serous macular detachment, macular holes, cystic changes in the macula, vision loss, and deterioration of the visual field, hence, requiring regular screening.[5]

Occurrence of optic disc pit in a patient with DLS has not been reported. These could be two different congenital anomalies of the eyes found incidentally in the same patient or there might be a possible unexplained association.
